# Clinical Utility of a Unique Genome-Wide DNA Methylation Signature for *KMT2A*-Related Syndrome

**DOI:** 10.3390/ijms23031815

**Published:** 2022-02-05

**Authors:** Aidin Foroutan, Sadegheh Haghshenas, Pratibha Bhai, Michael A. Levy, Jennifer Kerkhof, Haley McConkey, Marcello Niceta, Andrea Ciolfi, Lucia Pedace, Evelina Miele, David Genevieve, Solveig Heide, Mariëlle Alders, Giuseppe Zampino, Giuseppe Merla, Mélanie Fradin, Eric Bieth, Dominique Bonneau, Klaus Dieterich, Patricia Fergelot, Elise Schaefer, Laurence Faivre, Antonio Vitobello, Silvia Maitz, Rita Fischetto, Cristina Gervasini, Maria Piccione, Ingrid van de Laar, Marco Tartaglia, Bekim Sadikovic, Anne-Sophie Lebre

**Affiliations:** 1 Department of Pathology and Laboratory Medicine, Western University, London, ON N6A 3K7, Canada; aidin.foroutan@lhsc.on.ca (A.F.); shaghsh@uwo.ca (S.H.); 2 Verspeeten Clinical Genome Centre, London Health Sciences Centre, London, ON N6A 5W9, Canada; pratibha.bhai@lhsc.on.ca (P.B.); michael.levy@lhsc.on.ca (M.A.L.); jennifer.kerkhof@lhsc.on.ca (J.K.); haley.mcconkey@lhsc.on.ca (H.M.); 3 Genetics and Rare Diseases Research Division, Ospedale Pediatrico Bambino Gesù, IRCCS, 00146 Rome, Italy; marcello.niceta@opbg.net (M.N.); andrea.ciolfi@opbg.net (A.C.); marco.tartaglia@opbg.net (M.T.); 4 Department of Pediatric Onco-Hematology and Cell and Gene Therapy, Ospedale Pediatrico Bambino Gesù, IRCCS, 00146 Rome, Italy; lucia.pedace@opbg.net (L.P.); evelina.miele@opbg.net (E.M.); 5 Medical Genetic Department for Rare Diseases and Personalized Medicine, Reference Center AD SOOR, AnDDI-RARE, Groupe DI, Inserm U1183—Institute for Regenerative Medicine and Biotherapy, Montpellier University, Centre Hospitalier Universitaire de Montpellier, 34090 Montpellier, France; d-genevieve@chu-montpellier.fr; 6 Department of Genetics, Referral Center for Intellectual Disabilities, APHP Sorbonne University, Pitié Salpêtrière Hospital, 75013 Paris, France; solveig.heide@aphp.fr; 7 Department of Clinical Genetics, Amsterdam UMC, University of Amsterdam, 1105 AZ Amsterdam, The Netherlands; m.alders@amsterdamumc.nl; 8 Center for Rare Diseases and Congenital Defects, Fondazione Policlinico Universitario A. Gemelli, IRCCS, 00168 Rome, Italy; giuseppe.zampino@unicatt.it; 9 Facoltà di Medicina e Chirurgia, Università Cattolica del S. Cuore, 20123 Roma, Italy; 10 Department of Molecular Medicine and Medical Biotechnology, Università di Napoli “Federico II”, 80131 Naples, Italy; giuseppe.merla@unina.it; 11 Laboratory of Regulatory and Functional Genomics, Fondazione Casa Sollievo della Sofferenza, 71013 San Giovanni Rotondo, Italy; 12 Service de Génétique, CHU de Rennes, 35203 Rennes, France; melanie.fradin@chu-rennes.fr; 13 Medical Genetics Department, University of Angers, CHU Angers, 49000 Angers, France; bieth.e@chu-toulouse.fr; 14 Department of genetics, CHU d’Angers, 49000 Angers, France and MitoVasc, UMR CNRS 6015-INSERM 1083, University of Angers, 49055 Angers, France; dobonneau@chu-angers.fr; 15 CHU Grenoble Alpes, Inserm, U1209, Institute of Advanced Biosciences, Université Grenoble Alpes, 38000 Grenoble, France; kdieterich@chu-grenoble.fr; 16 Medical Genetics Department, Inserm U1211, Reference Center AD SOOR, AnDDI-RARE, Bordeaux University, Centre Hospitalier Universitaire de Bordeaux, 33076 Bordeaux, France; patricia.fergelot@chu-bordeaux.fr; 17 Service de Génétique Médicale—Institut de Génétique Médicale d’Alsace—Hôpitaux Universitaires de Strasbourg, 67091 Strasbourg, France; elise.schaefer@chru-strasbourg.fr; 18 Inserm, UMR1231, Equipe GAD, Bâtiment B3, Université de Bourgogne Franche Comté, 15 boulevard du Maréchal de Lattre de Tassigny, 21000 Dijon, France; laurence.faivre@chu-dijon.fr (L.F.); antonio.vitobello@u-bourgogne.fr (A.V.); 19 Unité Fonctionnelle Innovation en Diagnostic Génomique des Maladies Rares, FHU-TRANSLAD, Department of Medical Genetics, Dijon University Hospital, 21000 Dijon, France; 20 Clinical Pediatric Genetics Unit, Pediatrics Clinics, MBBM Foundation, S. Gerardo Hospital, 20900 Monza, Italy; silviabeatrice.maitz@eoc.ch; 21 Clinical Genetics Unit, Department of Pediatric Medicine, Giovanni XXIII Children’s Hospital, 02115 Bari, Italy; rfischetto@libero.it; 22 Medical Genetics, Department of Health Sciences, Università degli Studi di Milano, 20142 Milan, Italy; cristina.gervasini@unimi.it; 23 Department of Sciences for Health Promotion and Mother and Child Care “G. D’Alessandro”, University of Palermo, 90127 Palermo, Italy; maria.piccione@unipa.it; 24 Department of Clinical Genetics, Erasmus MC, University Medical Center Rotterdam, 3000 CA Rotterdam, The Netherlands; i.vandelaar@erasmusmc.nl; 25 Team Physiopathologie des Maladies Psychiatriques, GDR3557-Institut de Psychiatrie, Institute of Psychiatry and Neuroscience of Paris (IPNP), INSERM U1266, Université de Paris, 75006 Paris, France; 26 Centre Hospitalier Universitaire de Reims, Pôle de Biologie Médicale et Pathologie, Service de GénéTique, 51100 Reims, France

**Keywords:** epigenetics, DNA methylation, episignature, Wiedemann–Steiner syndrome, *KMT2A* gene, intellectual disability, neurodevelopmental disorders

## Abstract

Wiedemann–Steiner syndrome (WDSTS) is a Mendelian syndromic intellectual disability (ID) condition associated with hypertrichosis cubiti, short stature, and characteristic facies caused by pathogenic variants in the *KMT2A* gene. Clinical features can be inconclusive in mild and unusual WDSTS presentations with variable ID (mild to severe), facies (typical or not) and other associated malformations (bone, cerebral, renal, cardiac and ophthalmological anomalies). Interpretation and classification of rare *KMT2A* variants can be challenging. A genome-wide DNA methylation episignature for *KMT2A*-related syndrome could allow functional classification of variants and provide insights into the pathophysiology of WDSTS. Therefore, we assessed genome-wide DNA methylation profiles in a cohort of 60 patients with clinical diagnosis for WDSTS or Kabuki and identified a unique highly sensitive and specific DNA methylation episignature as a molecular biomarker of WDSTS. WDSTS episignature enabled classification of variants of uncertain significance in the *KMT2A* gene as well as confirmation of diagnosis in patients with clinical presentation of WDSTS without known genetic variants. The changes in the methylation profile resulting from *KMT2A* mutations involve global reduction in methylation in various genes, including homeobox gene promoters. These findings provide novel insights into the molecular etiology of WDSTS and explain the broad phenotypic spectrum of the disease.

## 1. Introduction

Wiedemann–Steiner syndrome (WDSTS, MIM# 605130) is a rare severe autosomal dominant disorder, characterized by intellectual disability (ID), developmental delay, hypertrichosis cubiti and distinctive facial features [1,2,3,4,5,6,7,8]. The phenotypic spectrum of WDSTS has recently been expanded with clinical features such as ocular abnormalities, recurrent infections of the genitourinary and/or respiratory tract, cardiac or urogenital malformations and skeletal abnormalities, including craniovertebral junction (CVJ) anomalies [6]. The WDSTS phenotype and genotype–phenotype correlation is currently not fully understood. Moreover, the mild/unusual WDSTS presentations may be challenging to be recognized [9].

The introduction of next-generation sequencing (NGS) and the implementation of human phenotype ontology (HPO) have revolutionized diagnostics for rare diseases [10]. Reverse dysmorphology, defined as the delineation of new syndromes primarily by genotype followed by the description of the phenotype, is now preferred [11,12]. While phenotypical evaluation of patients has still remained critical for the process of diagnosis, the clinical diagnosis for chromatin-related disorders is often established only after identification of a causative genetic variant [9].

WDSTS is caused by heterozygous pathogenic variants in the *KMT2A* (lysine methyltransferase 2A) gene (MIM# 159555), located on chr11q23, previously known as MLL (mixed lineage leukemia). Germline and somatic *KMT2A* variants are, respectively, associated with WDSTS and multiple neoplastic diseases, along with gene structural rearrangements that are common in acute leukemia [13]. KMT2A encodes a histone H3K4 methyltransferase enzyme that regulates chromatin mediated transcription and is widely expressed in most human tissues. KMT2A is involved in specific complexes mediating the methylation of lysine 4 of histone H3 (H3K4me) and acetylation of lysine 16 of histone H4 (H4K16ac), tags for epigenetic transcriptional activation [14,15,16]. KMT2A is essential for embryonic development, hematopoiesis, and neural development. Known targets include the homeobox (*HOX*) genes, a family of transcription factors essential for normal embryonic development [17,18].

KMT2A is a 3972 amino acid (aa) multidomain protein (NP_001184033.1), comprising three DNA-binding AT-hooks at the N-terminus, a cysteine-rich CXXC domain, a plant homeodomain (PHD) finger motif, a bromodomain, a transactivation domain (TAD), a FYRN domain, a WDR5 interaction (Win) motif, and a C-terminal SET domain [3]. The SET domain is involved in the histone monomethylation, dimethylation, or trimethylation activity of the protein [19,20,21]. Pathogenic variants in the *KMT2A* gene lead to defects in chromatin remodeling [15] and are thought to result in global changes in gene expression throughout development leading to abnormalities in multiple body systems. WDSTS or *KMT2A*-related syndrome is a typical epigenetic machinery disorder, included in chromatin-related disorders, a group of diseases caused by alterations in genes coding for components of the epigenetic apparatus [16]. The proteins associated with chromatin-related disorders act in concert to control the chromatin opening and closing thus regulating gene expression by modification (i.e., methylation, acetylation, etc.) of histones and DNA. Chromatin-related disorders frequently present with overlapping clinical features and inconclusive or ambiguous genetic findings which can confound accurate diagnosis and clinical management [9]. An expanding number of genetic syndromes have been shown to have unique genomic DNA methylation patterns or episignatures. Peripheral blood episignatures can be used for diagnostic testing and for classification of genetic variants. Recently, it has been shown that some diseases influencing DNA methylation have specific methylation signatures, referred to as episignatures [22,23,24,25]. Episignature analysis has recently been implemented as a diagnostic clinical genomic DNA methylation test, in individuals with rare disorders, providing strong evidence of its clinical utility including the ability to provide conclusive diagnostic findings in most subjects tested [26].

A consensus on the clinical classification of genomic variants based on the American College of Medical Genetics and Genomics (ACMG) criteria has recently been attained. WDSTS-associated variants are loss-of-function (LoF) and missense variants. In this study, we identified a unique genome-wide DNA methylation episignature for *KMT2A*-related syndrome. We compared it with episignatures obtained to a large cohort of patients with various episignature disorders within the EpiSign Knowledge Database (EKD) [22,26], including patients with neurodevelopmental syndromic disorders, especially with regard to Kabuki1, caused by pathogenic variants in *KMT2D* gene, as some patients with missense and splice site variants in *KMT2A* have been reported to show phenotype similarities to the ones observed in the Kabuki1 syndrome and because similar to *KMT2A*, *KMT2D* also mediates the methylation of lysine 4 of histone H3 [27]. Using in silico studies, aggregated, population and mutations-specific databases, and genome-wide DNA methylation signatures, we were able to definitively classify 56 *KMT2A* variants.

## 2. Results

### 2.1. Demographic and Molecular Characteristics of Patients

The molecular description at diagnosis and demographics of a cohort of 60 patients with clinical diagnosis for WDSTS is shown in Table 1. Fifty-six patients carried *KMT2A* intragenic variants (missense, nonsense, indel or splice site changes, including variants of uncertain significance (VUS)) and four patients had only a clinical diagnosis of WDSTS or Kabuki syndrome.

The molecular description at diagnosis and demographics of a cohort of 74 patients with clinical diagnosis for Kabuki is shown in Table S1. Sixty-six patients carried *KMT2D* intragenic pathogenic variants (missense, nonsense, indel or splice site variants) and eight patients did not have variant information. All patients had a clinical diagnosis of Kabuki syndrome associated with a Kabuki1 episignature.

### 2.2. Detection and Verification of an Episignature for WDSTS

**Figure 1:**
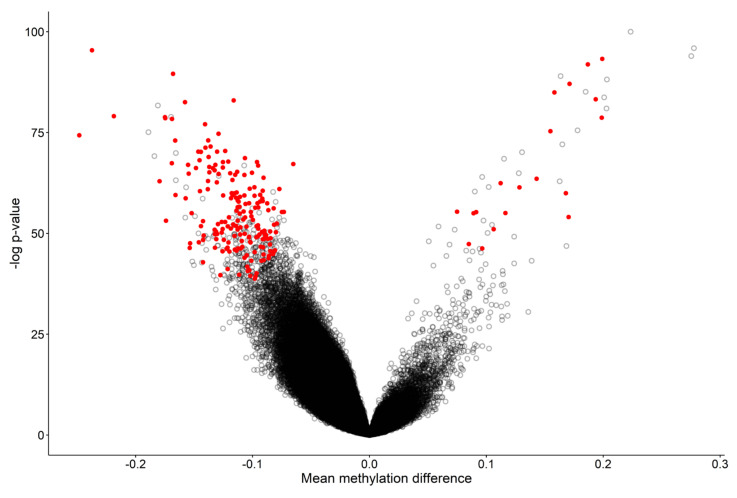
Volcano plot of methylation difference between 41 WDSTS samples and controls versus statistical significance (-log *p*-value) of individual probes. Red dots represent selected, significant differentially methylated probes (DMPs). Positive and negative mean methylation difference show hypermethylation and hypomethylation, respectively.

Forty-one WDSTS samples with pathogenic *KMT2A* variants (training set, Pt.1 to Pt.41 in Table 1) and 82 control samples were included for detection of an episignature for WDSTS syndrome. The changes in the methylation status driven by *KMT2A* pathogenic variants involve an overall (87%) global reduction in methylation (Figure 1).

**Figure 2:**
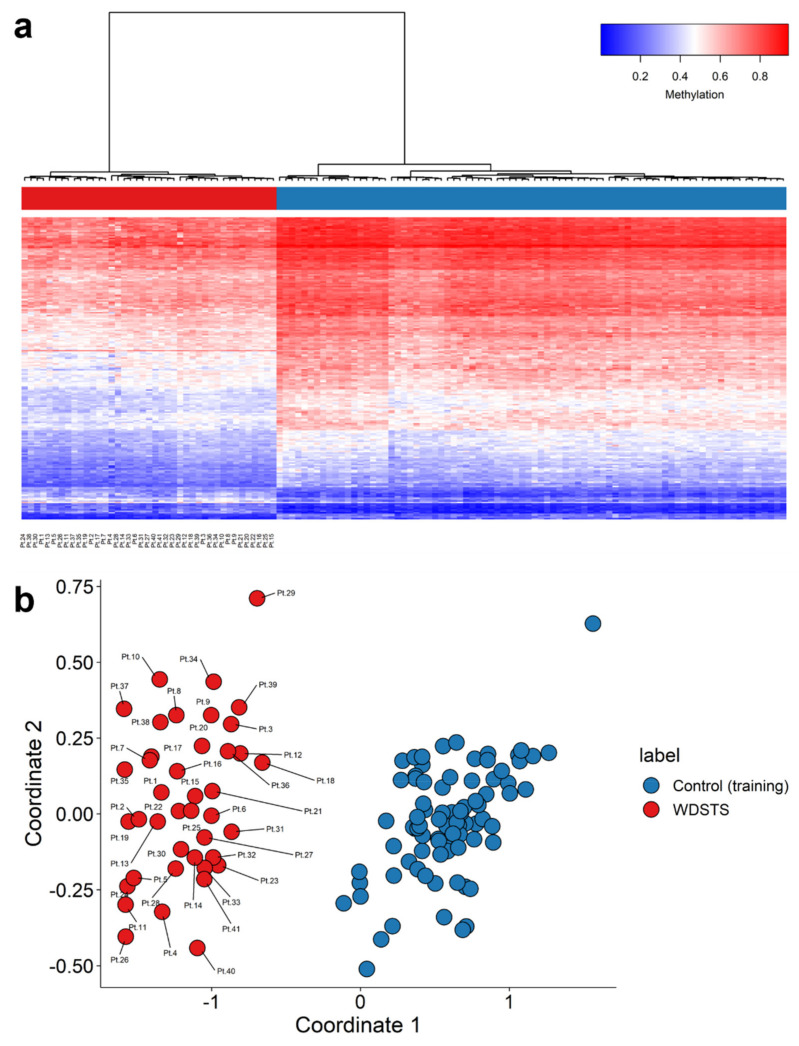
Identification of a WDSTS episignature. (**a**) Hierarchical clustering with Ward’s method on Euclidean distance was performed. In the heatmap plot, each row illustrates a selected CpG site, and each column is related to a sample. The heatmap color scale indicates the range of methylation level; from blue (no methylation or 0) to red (full methylation or 1). This plot conveys that the detected episignature clearly differentiates between 41 WDSTS samples and controls; (**b**) multidimensional scaling (MDS) plot using the selected probes. MDS plot illustrates power of the signature in separating the 41 WDSTS samples and control samples. Blue circles represent control subjects and red circles indicate subjects with pathogenic variants in the *KMT2A* gene and a confirmed diagnosis of the syndrome.

The 207 differentially methylated probes (DMPs) (Figure 1 and Table S2) selected using the three-step process described in the Methods section were used for the purpose of constructing unsupervised and supervised classification models. The methylation levels at these 207 CpG sites were considered as the identifying episignature of the syndrome. In order to assess the robustness of the episignature in differentiating between the case and control samples, hierarchical clustering (Figure 2a) and multidimensional scaling (MDS) analysis (Figure 2b) were performed, resulting in a clear separation between these two groups.

Forty-one rounds of cross-validation on MDS plot were performed using 40 WDSTS samples as the training set and a single WDSTS sample as the testing set. In all steps, the testing samples were correctly clustered with the training samples, further providing evidence of a robust common DNA methylation episignature (Figure S1).

### 2.3. Construction of the Binary Prediction Model

**Figure 3:**
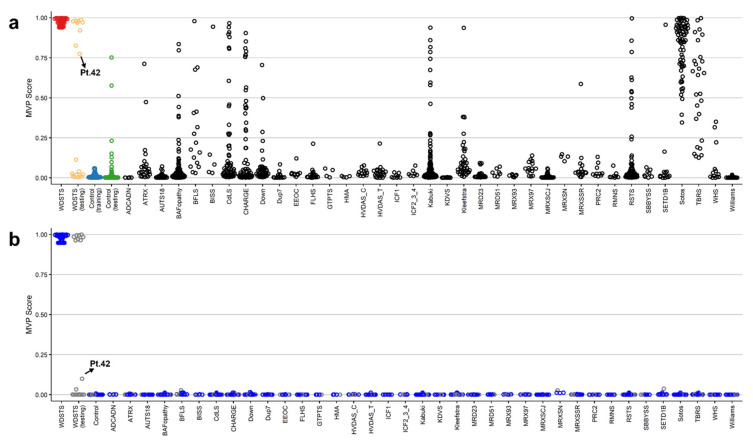
The methylation variant pathogenicity (MVP) scores plot. (**a**) The MVP scores were created by the SVM trained by comparing the 41 WDSTS samples against controls; (**b**) the MVP scores created by the SVM trained by comparing 41 WDSTS samples against controls and 38 neurodevelopmental disorders and congenital anomalies available in the EKD. The blue circles represent the training samples and the grey circles represent the testing samples.

Two methylation variant pathogenicity (MVP) plots were generated to confirm specificity of the classification model. In the first MVP plot where the support vector machine classifier (SVM) was trained by comparing the 41 WDSTS samples against controls, the classifier showed a high sensitivity for all WDSTS cases and nine WDSTS (testing) samples (including four samples without known *KMT2A* variants (Pt.42, Pt.44, Pt.45, Pt.50), one with a canonical -1 splice site variant (Pt.43), one with a nonsense variant (Pt.49), two out of 11 with a missense variant (Pt.46, Pt.47), and one out of two with an in-frame deletion (Pt.48)), with all samples scoring high on the MVP score axis (Figure 3a), further confirming the previous heatmap and MDS results. Some samples from control (testing) as well as other disease cohorts available in EKD (including BAFopathy complex, Börjeson–Forssman–Lehmann syndrome (BFLS), Cornelia de Lange syndrome (CdLS), CHARGE, Down, Kabuki (including Kabuki1 and Kabuki2), Rubinstein–Taybi syndrome (RSTS), Sotos, and Tatton–Brown–Rahman syndrome (TBRS) plus one sample from (Alpha thalassemia/mental retardation X-linked syndrome) ATRX, Blepharophimosis-impaired intellectual development syndrome SMARCA2 Syndrome (BISS), Kleefstra, Mental retardation, X-linked, Snyder–Robinson type (MRXSSR), and SETD1B cohorts) showed an elevated MVP score, suggesting level of similarity in the DNA methylation profiles between these disorders.

To increase the specificity of the classifier, an SVM was trained by comparing the 41 WDSTS cases against WDSTS (testing) samples, controls, as well as 38 neurodevelopmental disorders (NDDs) and congenital anomalies (CAs) with known episignatures present in the EKD. A high MVP score was seen for 41 WDSTS samples and eight WDSTS (testing) samples (including three out of four with no *KMT2A* variant information (Pt.44, Pt.45, Pt.50), one with a canonical −1 splice site variant (Pt.43), one with a nonsense variant (Pt.49), two out of 11 with a missense variant (Pt.46, Pt.47), and one out of two with an in-frame deletion (Pt.48)) along with much improved specificity relative to other EpiSign conditions (Figure 3b). Furthermore, one WDSTS (testing) sample with no variant information (Pt.42) had an MVP score of 0.10.

### 2.4. Validation of WDSTS Signature Using Testing Cohort and Comparison to Kabuki1 Samples

**Figure 4:**
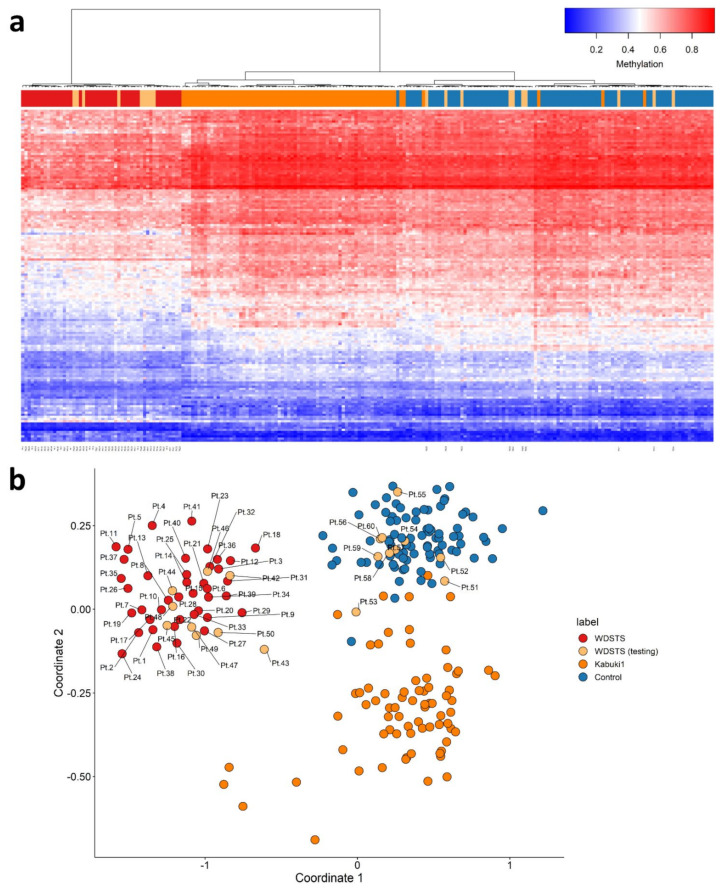
Adding WDSTS (testing) and Kabuki1 samples to the WDSTS signature. (**a**) Hierarchical clustering; (**b**) multidimensional scaling.

Nineteen WDSTS (testing) were used for validation of WDSTS signature, of which four had WDSTS or Kabuki phenotypes but without known *KMT2A* variants (Pt.42, Pt.44, Pt.45, Pt.50), one had a canonical -1 splice site variant (Pt.43), one had a nonsense variant (Pt.49), 11 had missense variants (Pt.46, Pt.47, Pt.51, Pt.52, Pt.53, Pt.54, Pt.55, Pt.57, Pt.58, Pt.59, Pt.60), and two had in-frame deletions (Pt.48, Pt.56). Interestingly, all four WDSTS (testing) samples without known *KMT2A* variants (Pt.42, Pt.44, Pt.45, Pt.50) were grouped with WDSTS samples (Figure 4). However, amongst the remaining 15 samples with known *KMT2A* variants, 10 (Pt.51 to Pt.60) and five (Pt.43 and Pt.46 to Pt.49) were grouped with control and WDSTS samples, respectively. In addition, using the WDSTS classifier, Kabuki1 samples showed segregation in relation to the control samples. As well, Kabuki1 samples were completely segregated from WDSTS samples, further confirming the results depicted in Figure 3b.

### 2.5. Identification of Differentially Methylated Regions

The identified WDSTS episignature was used to search for differentially methylated regions (DMRs), and resulted in identification of seven DMRs and 207 DMPs (Figure S2 and Table S3). Functional annotation clustering analysis of the DMPs was performed using DAVID [29], and resulted in identification of three significant clusters (Table S4) associated with major terms including (1) homeobox, DNA binding, transcription regulation; (2) regulation of transcription from RNA polymerase II promoter; and (3) T-box transcription factors genes. A relevant proportion of these genes contain hypomethylated regions. This analysis showed enrichment for pathways associated with development and transcription.

### 2.6. Definitive Classification of KMT2A Variants

The literature described patients already reported with confirmed clinical and molecular diagnoses of WDSTS. Clinical data were collected from literature [1,2,3,4,5,6,7] and accurate HPO terms were selected (Table S5). Using in silico studies, aggregated, population and mutations-specific databases, and genome-wide DNA methylation signatures, we definitively classified 54 *KMT2A* variants (including 13 missense variants) (Table S6). Variants information was uploaded in MobiDetails [30,31].

## 3. Discussion

Neurodevelopmental disorders, including the Fragile X and Rett syndromes, disorders of imprinting (such as Angelman and Prader–Willi syndromes), and also the Phelan-McDermid, Sotos, Kleefstra, Coffin Lowry, Kabuki, Charge and ATRX syndromes, are associated with aberrant epigenetic regulation of processes critical for normal brain development [22,26]. The diagnostic utility of genome-wide DNA methylation analysis using peripheral blood has been shown for patients with these NDDs. Over 43 NDDs are currently described with associated distinct DNA methylation episignatures [22,32]. Many of the related genes have a role in the epigenetic machinery such as DNA methylation, histone modification, or chromatin remodeling. Study of the DNA methylation episignatures has been useful to assign a diagnosis to patients with NDDs that remained unresolved by conventional testing or in patients with incorrect initial clinical diagnosis [23,25,26]. EpiSign is the first genome-wide DNA methylation clinical test for patients with NDDs which can be used to assess a clinical diagnostic assessment or to reclassify VUS variants [23].

The intellectual developmental disorder WDSTS was described as a syndromic condition in which ID is associated with hypertrichosis cubiti, short stature, and characteristic facies. Baer et al. described a broad phenotypic spectrum with regard to ID (mild to severe), the facies (typical or not of WDSTS) and associated malformations (bone, cerebral, renal, cardiac and ophthalmological anomalies) [2]. Hypertrichosis cubiti was supposed to be pathognomonic but was found only in 61% of the cases [2]. A majority of patients exhibited suggestive features, but others were less characteristic, only identified by molecular diagnosis [2,9]. The authors suggested that the prevalence of WDSTS is higher than expected in patients with ID, suggesting that *KMT2A* is a major gene in ID [2].

Following the identification of the causative *KMT2A* gene in 2012 [33], all types of variants, including missense variants in the *KMT2A* gene, were reported as the causal variants of the disorder. In this study, DNA methylation data were collected from peripheral blood of a patient cohort including 60 WDSTS patients, of which 55 had known *KMT2A* variants. The classification model for WDSTS syndrome was built with a training set (41 WDSTS patients) and a control set (82 matched control samples from EKD) using 207 DMPs (Figure 2 and Table S2). In addition, seven DMRs were also identified (Figure S2 and Table S3). The classification model was tested with a testing set (19 patients with uncertain clinical diagnosis) and allowed to reclassify 13 *KMT2A* missense variants as probably benign (ACMG class 4) or pathogenic (ACMG class 1) (Figure 3 and Figure S3, Table S6). Nine patients from the testing set were finally classified as WDSTS (including four samples without *KMT2A* variant information (Pt.42, Pt.44, Pt.45, Pt.50), one with a canonical -1 splice site variant (Pt.43), one with a nonsense variant (Pt.49), two out of 11 with a missense variant (Pt.46, Pt.47), and one out of two with an in-frame deletion (Pt.48)). Note that Pt.42 received a low MVP score while it was clustered with *KMT2A* training samples in both MDS plots and hierarchical clustering heatmap, even though with slightly different level of methylation for some probes in comparison with other *KMT2A* training samples. There should be something interesting about the Pt.42 but unfortunately no variant information or clinical details were available to make a solid conclusion about this difference. Then, the classification model was tested with a Kabuki1 set (74 patients) (Table S1). Kabuki1 samples were completely segregated from WDSTS samples (Figure 4).

KMT2A is a histone methyltransferase protein deemed as a positive global regulator of gene transcription. This protein belongs to the group of histone-modifying enzymes comprising transactivation domain 9aa TAD [34] and is involved in the epigenetic maintenance of transcriptional memory. Its role as an epigenetic regulator of neuronal function is an ongoing area of research. *KMT2A* gene encodes a transcriptional coactivator that plays an essential role in regulating gene expression during early development and hematopoiesis. The encoded protein contains multiple conserved functional domains. One of these domains, the SET domain, is responsible for its histone H3 lysine 4 (H3K4) methyltransferase activity which mediates chromatin modifications associated with epigenetic transcriptional activation. Enriched in the nucleus, the KMT2A enzyme mono, di and trimethylates H3K4 [35]. This protein is processed by the enzyme threonine aspartase 1 into two fragments [34,36] and regulates the transcription of specific target genes, including particular *HOX* genes during development [37,38].

The methylome analysis did highlight a substantial change in the global methylation pattern in WDSTS samples, and resulted in 87% hypomethylated and 13% hypermethylated probes. More detailed information about the 207 CpG probes selected in WDSTS episignature are summarized in Table S2. Full sensitivity and specificity of our model were illustrated in Figure 3b, where all case samples received a high MVP score and all control samples and individuals from the other 38 constitutional disorders and congenital anomalies received a score near zero. Methylation changes involved specific CpGs in regulatory regions, indicating a punctual effect on a relatively small subset of genes and cellular processes. Indeed, only 13% of the DMPs were represented by a hypermethylation change, indicating that the changes in the methylation status driven by *KMT2A* pathogenic variants concern a global tendency in a reduction in methylation.

Functional annotation clustering analysis of the 207 DMPs using DAVID identified three significant clusters (Table S4) associated with major terms including (1) homeobox, DNA binding, transcription regulation (i.e., *HOX* genes, *PRDM* genes, *ALX* genes, *SIX2*, *WT1*); (2) regulation of transcription from RNA polymerase II promoter (i.e., *HOX* genes, *ALX* genes, *SIX2*, *WT1*); and (3) T-box transcription factors genes (*TBX1*, *TBX2*, *TBX4*). A relevant proportion of these genes contain hypomethylated regions predominantly expressed in brain, but also in bones, kidney, heart, and eye. Among 207 DMPs, a distinctive hypomethylation pattern affecting genes from three of the four HOX clusters (*HOXA2*, *HOXA3*, *HOXA4*, *HOXA10*, *HOXB9*, *HOXC4*, *HOXC5*, *HOXC6*), the *MIR196A1* gene, two genes from the PRDM protein family (*PRDM14*, *PRDM16*), Homeobox protein aristaless-like genes (*ALX3* and *ALX4*), *SIX2*, T-box transcription factors genes (*TBX1*, *TBX2*, *TBX4*) and *WT1* gene were seen. Furthermore, a distinctive hypermethylation pattern affecting genes related to the HOX clusters (*HOXA6*, *HOXA7*, *HOXA9*, *HOXA10* and *PRDM16*) were seen.

In humans, the 39 *HOX* genes are arranged in four clusters (*HOXA*, *HOXB*, *HOXC*, and *HOXD*) in chromosomes 7p15, 17q21.2, 12q13, and 2q31, respectively. This highly conserved family belongs to the homeobox class of genes that encode transcription factors required for normal embryonic global development, including brain development [18] and embryology of the bony CVJ [17]. *HOX* genes function in multiple neuronal classes to shape synaptic specificity during development, suggesting a broader role in circuit assembly. They play key roles in defining the identity, organization, and peripheral connectivity of motor neuron subtypes, and their target effectors are beginning to be defined, the contribution of *HOX* genes to synaptic specificity in neural circuits within the central nervous system (CNS) remains to be resolved [39]. *HOX* genes are essentially absent in healthy adult brain, whereas they are detected in malignant brain tumors, namely gliomas [18]. In embryonic stem cells, which do not express *HOX* genes, whole HOX clusters are fully decorated by H3K27me3, while at their promoter area, this mark co-exists with H3K4me3, constituting the so-called bivalent chromatin. Deposition of H3K4me3 at HOX clusters in mammals relies on the COMPASS-like complexes that contains mammalian Set1 homologs (KMT2A to KMT2G proteins) and the homologues of Drosophila Trx [18,35]. Interestingly, this complex also contains the H3K27me3-demethylase KDM6A that removes H3K27me3 at HOX loci [18]. The dynamics of H3K27me3/H3K4me3 distribution along the different HOX clusters impacts their 3D architecture. The *PRDM14*, *PRDM16* and *MIR196A1* genes have been also linked functionally to HOX function. A gene-set enrichment analysis is discussed in additional data file. However, the methylation pattern was analyzed in leukocytes, which might considerably differ in neurons and in other cell lines.

Here, we provide a specific DNA methylation pattern in affected WDSTS patients. Patients with WDSTS which have *KMT2A* pathogenic variants have a distinct epigenetic signature in peripheral blood from a variety of other NDDs, including syndromes that may clinically overlap with WDSTS, like Kabuki type 1 patients with *KMT2D* pathogenic variants or Kabuki1 episignature (Figure 4). We demonstrate that WDSTS is characterized by an episignature, which is defined by a particular hypomethylation profile with respect to healthy subjects. WDSTS episignature is robust, enabling a discovery and validation of the highly sensitive and specific signal. Hypermethylation of homeobox gene promoters (including *HOX* genes), is emerging as a pan-cancer signature with patient-specific DNA methylation patterns [40]. Aberrant DNA methylation is a well-documented signature at HOX loci in glioma [18]. Our result suggests here global hypomethylation of various genes including homeobox gene promoters in WDSTS. *KMT2A* pathogenic variants may disturb the normal process of H3K4me3 deposition and H3K27me3 removal that are coupled at homeobox gene promoters.

Missense variants may present challenges for assessment of clinical impact on the protein function. In such cases, this WDSTS epigenetic classifier may help solve many clinically ambiguous cases presenting with a neurodevelopmental phenotype. Implementation of a routine genome-wide DNA methylation testing is suggested to be considered in the clinical management of patients with NDDs. The use of DNA obtained from peripheral blood samples makes this assay easily supported by diagnostic laboratories. DNA methylation profiling has the capability to detect episignatures from a variety of clinically related NDDs on the same array. It could be applied as an informative and cost-effective first-tier genetic diagnostic test for patients without prior molecular tests.

While methylation changes in DMRs suggest the possibility of gene expression modifications, further functional genomics analysis would be necessary to better understand the pathophysiology of these epigenetic changes. Investigation of genes affected by the abnormal DNA methylation may lead to the identification of novel targets for more personalized treatment approaches. Our studies reported that the expression of several homeobox containing genes (including *HOX* or *HOX*-related genes) is consistently altered in blood of WDSTS patients. Considering the critical functional roles and putative prognostic value of specific *HOX* genes in cancer, including in malignant glioma, and their complex molecular interactions with upstream regulators and downstream targets, it becomes clear that additional studies are necessary to better understand how *HOX* genes operate in glioma but also possibly in WDSTS, and whether they may be therapeutically explored in the clinics.

## 4. Materials and Methods

### 4.1. Study Cohort

This study included 60 individuals, of which 41 (labeled as WDSTS in Table 1) were used for the purpose of probe selection and construction of the classification model. All the samples and records were de-identified. Informed consent for use of the clinical information was obtained from the patients. This study was approved by the Western University Research Ethics Board (REB 106302) and Ospedale Pediatrico Bambino Gesù Ethical Committee (1702_OPBG_2018). This and all other study procedures complied with the Declaration of Helsinki and French legislation and regulations.

### 4.2. Methylation Experiment and Selection of Matched Control Subjects

DNA samples extracted from peripheral blood were supplied to Illumina Infinium methylation EPIC (EPIC) bead chip arrays as well as Illumina Infinium HumanMethylation450 (450 K) BeadChip arrays followed by bisulfite conversion, and the methylation analysis was performed at the Western University and Ospedale Pediatrico Bambino Gesù (samples pt. 34–39) in accordance with the manufacturer’s protocol. The 450 K and EPIC arrays cover >450,000 and >850,000 human genomic CpG sites, respectively, which include 99% of RefSeq genes and 96% of CpG islands. The obtained methylated and unmethylated signal intensities were imported into R 4.0.3 for analysis. Normalization was performed according to the Illumina normalization method with background correction using the minfi package [41]. Probes that were located on the X and Y chromosomes, had a detection *p*-value >0.01, known to contain a SNP at or near CpG interrogation sites, or known to cross-react with other genomic regions were removed, in order to ensure that the difference observed between the two groups is solely based on methylation changes rather than other potentially confounding factors. Where indicated, sex and age of the DNA specimens were predicted using the minfi package (based on the median signal intensities of the probes on the X and Y chromosomes) and the wateRmelon package [42], respectively. Principal component analysis (PCA) was performed in order to observe the overall structure of the batches, as well as to identify outliers. Forty-one WDSTS samples having pathogenic variants in the *KMT2A* gene with confirmed diagnosis of the syndrome (labelled as WDSTS) were used as the case training set. Eighty-two (case to control ratio of 1:2) age, sex, and array type-matched control samples were selected from the EKD [22] using the MatchIt package [43,44] as the control training set. We performed a PCA subsequent to every matching round to detect outliers and examine the data structure, and removed outlier samples as well as samples with irregular data structure at each trial. This process was iterated until no outlier was observed in the first two components of the PCA.

### 4.3. DNA Methylation Profiling of WDSTS Syndrome

The procedure of differentially methylated probe selection was performed in accordance with previously published articles [22,45]. Methylation levels, called β values, for each probe were calculated as the ratio of methylated signal intensity to the sum of methylated and unmethylated signal intensities. These values were then converted to M values using logit transformation by the formula log2 (β/(1-β)) in order to obtain homoscedasticity for use in linear regression. Using the limma package [46], we performed linear regression and moderated the obtained *p*-values with the eBayes function. The DMPs from the comparison between case and control groups were selected in the following three-step process. First, 500 probes with the highest product of methylation difference means between the two groups and the negative of the logarithm of multiple-testing corrected *p*-values derived from the linear modeling by Benjamini–Hochberg (BH) method were selected. Subsequently, a receiver’s operating characteristic (ROC) curve analysis was performed and 250 probes with the highest area under the ROC curve (AUROC) were retained. Finally, those probes with a pair-wise correlation >0.85, within the case and control samples separately, which was measured using Pearson’s correlation coefficients were removed. This resulted in identification of 207 probes, which were considered as the DNA methylation signature for the WDSTS. In order to examine the robustness of this episignature in differentiating between case and control samples, unsupervised models were applied on the 207 DMPs. They include hierarchical clustering which was performed using Ward’s method on Euclidean distance as well as MDS analysis which was performed by scaling of the pair-wise Euclidean distances between samples. Then, 41 rounds of cross-validation were performed on MDS plot from the 41 WDSTS samples, of which 40 samples were used as the training set and a single sample was used as the testing set at each round.

### 4.4. Construction of a Classification Model for WDSTS Syndrome

Using the selected DMPs, a binary SVM with linear kernel was constructed by the e1071 package as described previously [22,23,47]. In order to obtain the best hyperparameter (cost) and to assess the accuracy of the classifier, a 10-fold cross-validation was performed during training. In 10-fold cross-validation, at each round, 90% of the samples were used for training and the remaining samples for testing. The model provides an MVP score, ranging from 0 to 1, for each sample. Scores near 1 indicate a high similarity between the methylation profile of that sample and that of the identified episignature, while scores near 0 indicate a low similarity. In order to evaluate the specificity of the classifier, more than 1700 samples with other neurodevelopmental syndromes from the EKD [22] were added to the model.

### 4.5. Classification of Kabuki1 Samples with Pathogenic KMT2D Variants as well as WDSTS (Testing) Samples

To assess the similarity of the methylation profiles of 74 Kabuki1 samples (Table S1) [22] and perform episignature analysis and variant classification in 19 WDSTS (testing) samples, both hierarchical clustering and MDS analysis were reconstructed using the initial WDSTS and 82 control samples as the training set, plus the 19 WDSTS (testing) and 74 Kabuki1 samples as the testing set.

### 4.6. Identification of the Differentially Methylated Regions of WDSTS Syndrome

In order to identify the regions that are differentially methylated between the subjects with WDSTS and controls, we used the DMRcate package [48] and selected regions containing a minimum of 3 CpG sites within 1 kb with at least 10% methylation difference between the case and control groups and a Fisher’s multiple comparison *p*-value < 0.01.

## 5. Conclusions

In conclusion, the identified WDSTS DNA methylation episignature is added to the list of Mendelian NDDs with known DNA methylation episignatures that can be used for screening and diagnosis of NDD patients. *KMT2A* pathogenic variants may disturb the normal process of H3K4me3 deposition coupled at gene promoters. The methylome analysis did highlight a substantial change in the global methylation pattern in WDSTS samples, and resulted in 87% hypomethylated and 13% hypermethylated probes. Our studies reported that the expression of several homeobox containing genes (including *HOX* or *HOX*-related genes) is consistently altered in the blood of WDSTS patients. If also present in other tissues, dysregulation of normal methylation of homeobox gene expression may explain part of the ID, facies and associated malformations observed in WDSTS patients. These provide novel insights into the molecular etiology of WDSTS and likely explain the broad phenotypic spectrum of the disease.

## Figures and Tables

**Table 1:** Demographic and molecular characteristics of WDSTS cohort.

ID	Sex	Age	Genetic Change in the *KMT2A* Gene (NM_001197104.2 ^§^)	Cohort_Array Type
Pt.1	m	2	c.5312G > A, p.(Trp1771 *)	WDSTS_EPIC
Pt.2	m	13	c.2647G > T, p.(Glu883 *)	WDSTS_EPIC
Pt.3	f	29	c.3635-1G > A, p.?	WDSTS_EPIC
Pt.4	m	5	c.9068del, p.(Gln3023Argfs *3)	WDSTS_EPIC
Pt.5	m	9	c.5572C > T, p.(Arg1858 *)	WDSTS_EPIC
Pt.6	f	4.5	c.9001del, p.(His3001Thrfs * 15)	WDSTS_EPIC
Pt.7	m	3	c.3464G > A, p.(Cys1155Tyr)	WDSTS_EPIC
Pt.8	m	6	c.3740_3741del, p.(Ser1247Cysfs * 12)	WDSTS_EPIC
Pt.9	f	4	c.3790C > T, p.(Arg1264 *)	WDSTS_EPIC
Pt.10	m	10	c.5251A > T, p.(Lys1751 *)	WDSTS_EPIC
Pt.11	m	12	c.3634 + 1G > A, p.?	WDSTS_EPIC
Pt.12	f	7	c.10837C > T, p.(Gln3613 *)	WDSTS_EPIC
Pt.13	m	12	c.3895_3896del, p.(Ser1299Profs * 26)	WDSTS_EPIC
Pt.14	m	13	c.478C > T, p.(Arg160 *)	WDSTS_EPIC
Pt.15	m *	21.8 ^#^	c.6735dup, p.(Val2246Serfs *2)	WDSTS_EPIC
Pt.16	m *	4 ^#^	c.2318_2319del, p.(Pro773Leufs * 12)	WDSTS_EPIC
Pt.17	f *	3.9 ^#^	c.3460C > T, p.(Arg1154Trp)	WDSTS_EPIC
Pt.18	f *	23.7 ^#^	c.8532_8533del, p.(Cys2844Trpfs * 24)	WDSTS_EPIC
Pt.19	m *	14.3 ^#^	c.11001dup, p.(Pro3668Thrfs * 8)	WDSTS_EPIC
Pt.20	f *	26.5 ^#^	c.2605G > T, p.(Glu869 *)	WDSTS_EPIC
Pt.21	m *	15.2 ^#^	c.10498C > T, p.(Gln3500 *)	WDSTS_EPIC
Pt.22	m *	17.2 ^#^	c.7630G > T, p.(Glu2544 *)	WDSTS_EPIC
Pt.23	m *	6.1 ^#^	c.10900 + 1G > A, p.?	WDSTS_EPIC
Pt.24	m *	1.1 ^#^	c.4256G > A, p.(Gly1419Asp)	WDSTS_EPIC
Pt.25	m *	9.1 ^#^	c.1539del, p.(Ile515Phefs * 52)	WDSTS_EPIC
Pt.26	m	17.6 ^#^	c.3460C > T, p.(Arg1154Trp)	WDSTS_EPIC
Pt.27	m	25.7 ^#^	c.2318dup, p.(Ser774Valfs * 12)	WDSTS_EPIC
Pt.28	m	67	c.5431C > T, p.(Arg1811 *)	WDSTS_EPIC
Pt.29	f	10	c.1128dup, p.(Gln377Thrfs * 12)	WDSTS_EPIC
Pt.30	f	1.9 ^#^	c.7975C > T, p.(Arg2659 *)	WDSTS_EPIC
Pt.31	f	34.9 ^#^	c.9538_9539del, p.(Ile3180Glnfs * 55)	WDSTS_EPIC
Pt.32	m	15.7 ^#^	c.7438C > T, p.(Arg2480 *)	WDSTS_EPIC
Pt.33	m	10 ^#^	c.3301C > T, p.(Arg1101 *)	WDSTS_EPIC
Pt.34	m	14	c.4727dup, p.(Tyr1576 *)	WDSTS_EPIC
Pt.35	m	19	c.3629_3634 + 1del, p.(Lys1211_Ala1212del)	WDSTS_EPIC
Pt.36	m	27	c.1821_1825del, p.(Arg608Ilefs * 9)	WDSTS_EPIC
Pt.37	m	22	c.3451C > T, p.(Arg1151 *)	WDSTS_EPIC
Pt.38	m	7	c.7150C > T, p.(Gln2384 *)	WDSTS_EPIC
Pt.39	f	2	c.7324G > T, p.(Glu2442 *)	WDSTS_EPIC
Pt.40	f	3	c.10736del, p.(Leu3580 *)	WDSTS_EPIC
Pt.41	m	17	c.4018G > T, p.(Glu1340 *)	WDSTS_EPIC
Pt.42 ^¥,₢^	m	19.5 ^#^	Not available	WDSTS (testing)_ 450k
Pt.43 ^¥^	f	9.1 ^#^	c.5803-1G > A, p.?^+^	WDSTS (testing)_ 450k
Pt.44 ^₢^	m	13	Not available	WDSTS (testing)_ 450k
Pt.45 ^₢^	f	2	Not available	WDSTS (testing)_ 450k
Pt.46	m	21	c.5806T > C, p.(Cys1936Arg)	WDSTS (testing)_EPIC
Pt.47	f	2	c.4426T > G, p.(Cys1476Gly)	WDSTS (testing)_EPIC
Pt.48	m	4	c.4432_4434del, p.(Arg1478del)	WDSTS (testing)_EPIC
Pt.49	f	2	c.4171C > T, p.(Gln1391 *)	WDSTS (testing)_EPIC
Pt.50 ^€,₢^	m	6.5 ^#^	Not available	WDSTS (testing)_ 450k
Pt.51 ^¥^	m	8.5 ^#^	c.3019G > T, p.(Gly1007Cys)^+^	WDSTS (testing)_ 450k
Pt.52	m	3	c.9575A > C, p.(Gln3192Pro)	WDSTS (testing)_EPIC
Pt.53	M *	9.4 ^#^	c.29C > T, p.(Pro10Leu)	WDSTS (testing)_EPIC
Pt.54	f	7	c.8545C > G, p.(Pro2849Ala)	WDSTS (testing)_EPIC
Pt.55	m	2	c.352G > T, p.(Val118Phe)	WDSTS (testing)_EPIC
Pt.56	f	3	c.11347_11376del, p.(Phe3783_Pro3792del)	WDSTS (testing)_EPIC
Pt.57	m	10	c.8387G > T, p.(Gly2796Val)	WDSTS (testing)_EPIC
Pt.58	m	2	c.100C > G, p.(Arg34Gly)	WDSTS (testing)_EPIC
Pt.59	f	25	c.10315_10316delinsAC, p.(Gly3439Thr)	WDSTS (testing)_EPIC
Pt.60	m	2	c.3379C > T, p.(Pro1127Ser)	WDSTS (testing)_EPIC

^¥^ Downloaded from the Gene Expression Omnibus (GEO) database: GSE116300 [27]; ^₢^ Pt.42 had a Kabuki syndrome phenotype, and Pt. 44, Pt. 45, and Pt.50 had WDSTS syndrome phenotypes; ^€^ downloaded from the Gene Expression Omnibus (GEO) database: GSE89353 [28]; ^§^ MANE Select/Ensembl canonical transcript; * sex was predicted using minfi package; ^#^ age was predicted using wateRmelon package; ^+^ reported as de novo variant in a patient with clinically defined Kabuki syndrome [27].

## Data Availability

Availability of data and materials Clinical data of the study cohort, probes defining the methylation episignature associated with *KMT2A* variants and list of regions differentially methylated in WDSTS are reported in additional files. Additional data are available from the corresponding authors upon request.

## Supplementary Materials

The following supporting information can be downloaded at: https://www.mdpi.com/article/10.3390/ijms23031815/s1.

## Abbreviations

WDSTS: Wiedemann–Steiner syndrome; ID: intellectual disability; SVM: support vector machine classifier; VUS: variant(s) of uncertain significance; HPO: Human Phenotype Ontology; CVJ: craniovertebral junction; NGS: next-generation sequencing; *KMT2A*: Lysine Methyltransferase 2A; MLL: Mixed Lineage Leukemia; H3K4me: methylation of lysine 4 of histone H3; H4K16ac: acetylation of lysine 16 of histone H4; aa: aminoacids; PHD: plant homeodomain; TAD: transactivation domain; ACMG: American College of Medical Genetics and Genomics; LoF: loss-of-function; EKD: EpiSign Knowledge Database; DMP: differentially methylated probes; MDS: multidimensional scaling; MVP: methylation variant pathogenicity; BAF: BRG1/BRM-associated factor; BFLS: Börjeson–Forssman–Lehmann syndrome; CdLS: Cornelia de Lange syndrome; RSTS: Rubinstein–Taybi syndrome; TBRS: Tatton–Brown–Rahman syndrome; ATRX: Alpha thalassemia/mental retardation X-linked syndrome; BISS: Blepharophimosis-impaired intellectual development syndrome SMARCA2 Syndrome; MRXSSR: Mental retardation, X-linked, Snyder–Robinson type; DMR: differentially methylated region; NDD: Neurodevelopmental disorders; CpG: Cytosine-phosphate-Guanine; CNS: central nervous system; PRDI-BF1: Positive Regulatory Domain I-Binding Factor1; RIZ1: Retinoblastoma protein-Interacting Zinc finger gene 1; HMT: histone methyl-transferases; PCA: principal component analysis; BH: Benjamini–Hochberg; ROC: receiver’s operating characteristic; AUROC: area under the ROC curve.
