# Supplementary material for: Clinical Utility of a Unique Genome-Wide DNA Methylation Signature for KMT2A-Related Syndrome
Source: Int J Mol Sci. 2022 Feb 5;23(3):1815. doi: 10.3390/ijms23031815 (PMC8836705; doi:10.3390/ijms23031815)
Supplement: Supplementary file 1 [file ijms-23-01815-s001.zip › ijms-1541287-supplementary materials/Supplementary Files/Additional data file.pdf]

Supplemental data

PRDM14, *PRDM16* and *MIR196A1* genes have been also linked functionally to HOX function. PRDM14 and PRDM16 proteins belong to the PRDM protein family and contain several zinc finger domains and a PR [PRDI-BF1 (Positive Regulatory Domain I-Binding Factor1) and RIZ1 (Retinoblastoma protein-Interacting Zinc finger gene 1) homologous domain] domain, which is related to the SET domain present in histone methyl-transferases (HMT). An HMT activity has been reported for several PRDM family members (including PRDM16). PRDM14 has been shown to interact with and decrease the stability and activity of the *HOXA1* transcription factor [38]. PRDM16 activity determines the production of mature neurons and their specific positions in the neocortex [39]. *In vitro* studies with brown adipocytes revealed that siRNA knockdown of *HOXA5* significantly decreased expression levels of *PRDM16* mRNA and protein [40].

MicroRNA-196 encoded by *MIR196A1* gene regulates *HOX* genes (*HOXA7, HOXB7, HOXC8* and *HOXB8)* expression in human gluteal adipose tissue [41]. We report here the aberrant methylation of 14 of the *HOX* genes or *HOX*-related genes (*HOXA2, A3, A4, A6, A7, A9, A10, B9, C4, C5, C6*, *MIR196A1, PRDM14* and *PRDM16*) in WDSTS patients. Notice that aberrant methylation of two other *HOX* genes (*HOXA4* and *A5*) was also described in Kabuki1 patients[42]. Dysregulation of normal methylation of *HOX* genes expression may explain part of the intellectual disability, the cerebral malformations and the CVJ anomalies.

Homeobox protein aristaless-like 3 and 4 (*ALX3* and *ALX4)* pathogenic gene variants are associated with Frontonasal dysplasia 1 and 2 (MIM#: 136760; 613451) and in Parietal foramina 2 (MIM#: 609597). *ALX3* and *ALX4* genes are involved in embryonic skeletal system (cranial skeleton, forelimb, hindlimb, digits) morphogenesis. *ALX4* gene is involved in hair follicle development. *SIX2* pathogenic gene deletion have also been associated with Frontonasal dysplasia [43].

Transcriptome profiling after deletion of *KMT2A* gene in frontal cortical neurons revealed decreased promoter-bound H3K4me3 peaks at various genes [44]. Among them was *MEIS2*, a homeobox transcription factor critical for development of forebrain neurons [45]. We report here the aberrant methylation of Homeobox containing genes including *MEIS2, CDX2, DLX4, PITX1, VAX2* and *VENTX* genes. *MEIS2, DLX4* and *PITX1* pathogenic gene variants are associated with rare diseases associated with cleft palate (MIM# 616788), cardiac defects and mental retardation (MIM#: 600987), or affecting bones (MIM#:119800; 186550).

T-box transcription factor 1, 2 and 4 (*TBX1, TBX2* and *TBX4)* pathogenic gene variants are associated with conotruncal anomaly face syndrome (CAFS) (MIM#217095), Vertebral anomalies and variable endocrine and T-cell dysfunction (MIM#: 618223), Ischiocoxopodopatellar syndrome with or without pulmonary arterial hypertension (MIM#: 147891) and Amelia, posterior, with pelvic and pulmonary hypoplasia syndrome (MIM#: 601360).

WT1 pathogenic gene variants are associated with Denys-Drash syndrome (MIM#: 194080), Frasier syndrome (MIM#: 136680) and Nephrotic syndrome, type 4 (MIM#: 256370).
