# Supplementary material for: Clinical Utility of a Unique Genome-Wide DNA Methylation Signature for KMT2A-Related Syndrome
Source: Int J Mol Sci. 2022 Feb 5;23(3):1815. doi: 10.3390/ijms23031815 (PMC8836705; doi:10.3390/ijms23031815)
Supplement: Supplementary file 1 [file ijms-23-01815-s001.zip › ijms-1541287-supplementary materials/Supplementary Files/Figure S2.pdf]

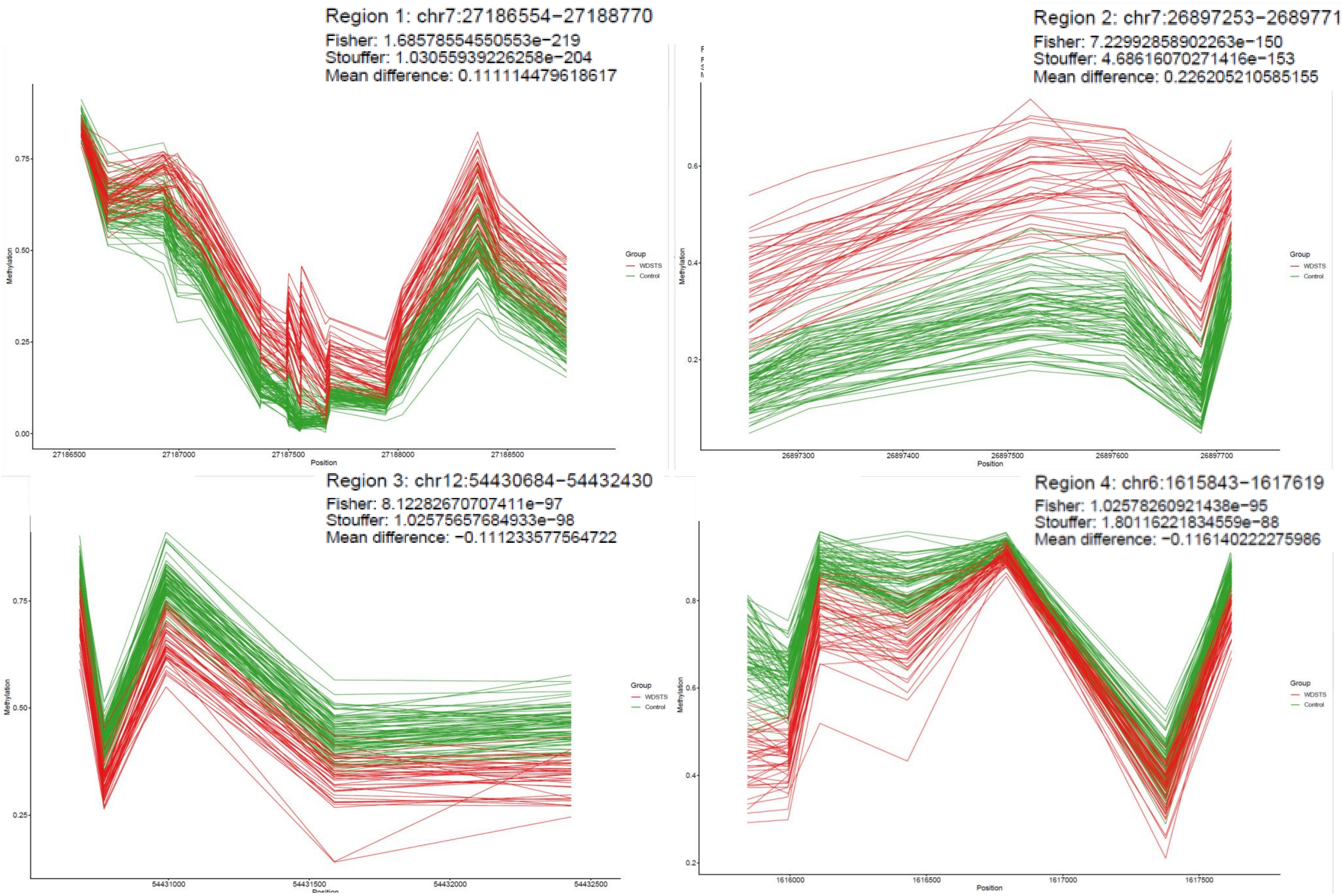


**Figure S2.** Four of the significant differentially methylated regions (DMRs) identified in the *KMT2A*-related group analysis.
