# Supplementary material for: Clinical Utility of a Unique Genome-Wide DNA Methylation Signature for KMT2A-Related Syndrome
Source: Int J Mol Sci. 2022 Feb 5;23(3):1815. doi: 10.3390/ijms23031815 (PMC8836705; doi:10.3390/ijms23031815)
Supplement: Supplementary file 1 [file ijms-23-01815-s001.zip › ijms-1541287-supplementary materials/Supplementary Files/Figure S3.pdf]

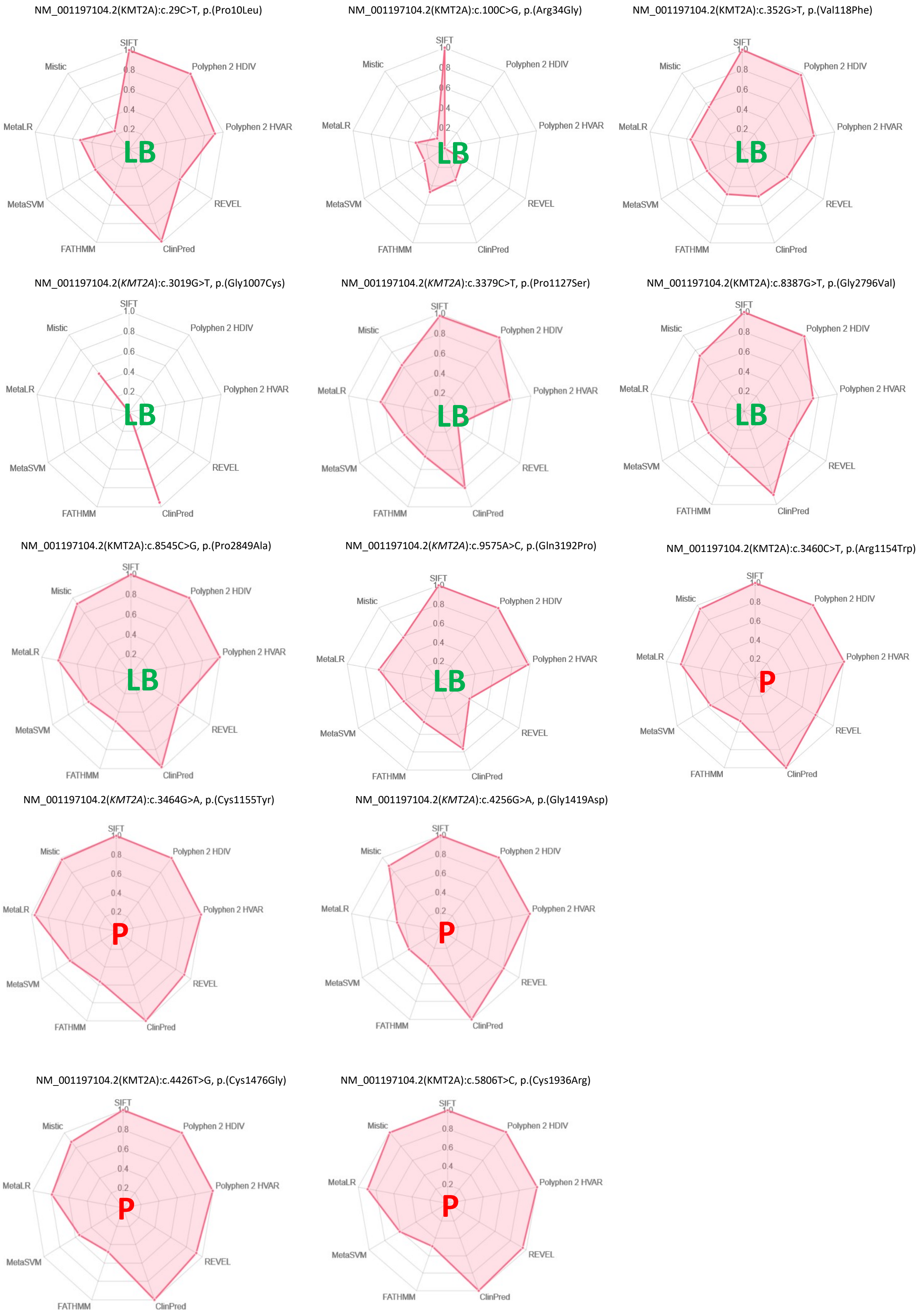


**Figure S3.** Radar view of missense predictors generated with Mobidetails (Values are normalized (0-1), 0 being the less damaging and 1 the most for each predictor)-2: Variants re-classified as likely benign (LB) (e.g. ACMG class 2) or pathogenic (P) (e.g. ACMG class 5).
